# The Rice R2R3-MYB Transcription Factor OsMYB55 Is Involved in the Tolerance to High Temperature and Modulates Amino Acid Metabolism

**DOI:** 10.1371/journal.pone.0052030

**Published:** 2012-12-14

**Authors:** Ashraf El-kereamy, Yong-Mei Bi, Kosala Ranathunge, Perrin H. Beatty, Allen G. Good, Steven J. Rothstein

**Affiliations:** 1 Department of Molecular and Cellular Biology, University of Guelph, Guelph, Ontario, Canada; 2 Department of Biological Sciences, University of Alberta, Edmonton, Alberta, Canada; 3 Horticulture Department, Faculty of Agriculture Ain Shams University, Cairo, Egypt; Max Planck Institute for Chemical Ecology, Germany

## Abstract

Temperatures higher than the optimum negatively affects plant growth and development. Tolerance to high temperature is a complex process that involves several pathways. Understanding this process, especially in crops such as rice, is essential to prepare for predicted climate changes due to global warming. Here, we show that *OsMYB55* is induced by high temperature and overexpression of *OsMYB55* resulted in improved plant growth under high temperature and decreased the negative effect of high temperature on grain yield. Transcriptome analysis revealed an increase in expression of several genes involved in amino acids metabolism. We demonstrate that OsMYB55 binds to the promoter regions of target genes and directly activates expression of some of those genes including glutamine synthetase (*OsGS1;2)* glutamine amidotransferase (*GAT1*) and glutamate decarboxylase 3 (*GAD3*). OsMYB55 overexpression resulted in an increase in total amino acid content and of the individual amino acids produced by the activation of the above mentioned genes and known for their roles in stress tolerance, namely L-glutamic acid, GABA and arginine especially under high temperature condition. In conclusion, overexpression of OsMYB55 improves rice plant tolerance to high temperature, and this high tolerance is associated with enhanced amino acid metabolism through transcription activation.

## Introduction

Plants are subject to various stress conditions during their life cycles that may adversely affect their productivity. An increase in average global temperature by 0.5°C over the 20^th^ century has been observed, with a further 2–4.5°C increase expected by the end of this century [1]. Productivity will be reduced for plants grown under various stress conditions. For instance, heat stress affects cotton seed germination [2], wheat coleoptiles growth [3], rice male sterility and spikelet fertility [4], [5], rice grain filing [6], [7] and apple fruit colour [8]. Rice yields decreased in correlation to the global increase in night temperature at the International Rice Research Institute farm between 1992 and 2003 [9].

Enhancing plant heat tolerance through genetic engineering requires the identification and characterization of the genes involved in the heat stress response. Most of the heat response studies in plants have been focused on the heat shock transcription factor (HSF) and heat shock proteins (HSP), reported to be induced during heat stress and involved in protecting the plant cell from negative heat effects [10]. The HSF activates the transcription of the HSP, which in turn act as molecular chaperones helping the plant cell to resolubilize protein aggregates after heat stress [11]. However it is hard to achieve thermotolerance by overexpression of a single HSP due to the complexity of the many interrelated pathways involved in the heat stress responses. These pathways include those involved in plant hormone response and sugar metabolism as well as a variety of other genes [12]–[14]. Therefore, other pathways besides the HSP could be potentially manipulated to enhance plant heat tolerance. For example, leaf soluble proteins, proline and soluble sugars are important factors and adaptive components in heat tolerant cotton cultivars [2]. Chemical treatments such as calcium and brassinosteroids have been reported to alleviate the negative effect of high temperature by altering the photosynthetic and antioxidative systems [3], [15]. In addition, amino acids such as GABA and arginine have been reported to have a role in heat stress [2]. Exogenous application of arginine resulted in reducing the negative effect of high temperatures on the productivity of wheat plants [16]. Further, genetic manipulation of genes other than HSF or HSP have been reported to enhance heat tolerance in plants, such as the constitutive expression of the maize *GASA4* genes [17], involved in ROS scavenging system [18], [19], glycinebetaine osmolyte synthesis genes [20], [21] and the genes involved in lipid metabolism [22], [23]. In addition, a transcriptome analysis of wheat revealed the induction of a large number of transcription factors following heat treatment, including 53 members of the MYB gene family [24].

The MYB plant transcription factor family members regulate numerous processes during the plant life cycle and are also involved in response to various environmental stresses [25]. The MYB proteins are classified into three major groups based on the number of adjacent repeats in the binding domain; R1R2R3-MYB, R2R3-MYB, and R1-MYB. Most plant MYB proteins are of the R2R3 type [26]–[28]. The R2R3-MYBs are involved in a wide range of physiological responses such as regulation of the isopropanoid and flavonoid pathways [29]–[30], control of the cell cycle, root growth (Mu et al. 2009) [31], and various defense and stress responses [32]–[35]. Despite the large number of genes in this family, the *Arabidopsis* MYB68 is the only member proposed to have a role in Arabidopsis heat tolerance [36]. The mutant of this gene showed reduced growth and higher lignin levels in the roots under high temperature, although it is not fully characterized. Until this present study, no rice MYB gene involved in heat tolerance had been identified.

In this study, we demonstrate that *OsMYB55* can enhance the vegetative growth and improve grain yield of rice growth under high temperature conditions. Molecular and biochemical analysis revealed that *OsMYB55* enhances amino acid metabolic pathways crucial for normal plant growth and development under high temperature.

## Materials and Methods

### Plant Materials and Growth Condition

All the experiments were carried out under growth cabinet conditions (Conviron, Manitoba, Canada). Rice seeds (*Oryza sativa* L. Kaybonnet) were planted in pots containing either Turface, a 100% baked calcined clay growth media with grain size between 2.5 and 3.5 mm (Turface MVP; Profile Products LLC, Buffalo Grove, IL, USA), or 75% vermiculite and 25% peat moss (SunGro Horticulture Canada Ltd., BC, Canada). Plants were grown in a full nutrient condition by using 1 g of the Nutricote® Total slow release fertilizer contains N-P-K, 13-13-13 supplemented with micronutrients (Chisso - Asahi Fertilizer CO. LTD. Tokyo, Japan) to each 500 ml pot. Eighteen plants were placed in a tray without holes and the water level was maintained by adding the water daily. Shortly after germination (10 days from planting) at moderate temperature condition, under 29°C and 23°C day/night temperatures, the pots contain the plants were divided into two treatments and subjected either to moderate temperature (29°C and 23°C day/night) or heat stress conditions (35°C and 26°C day/night) for four weeks. This treatment was followed by moving all plants to the moderate temperature until harvest. Plants subjected to long day conditions were grown at 16 hr light (∼500 µmol m^−2^s^−1^) and 8 hr dark, while in the neutral day condition, plants were subjected to 12 hr light (∼500 µmol m^−2^s^−1^) and 12 hr dark. Tissues were collected from the plants growing under the different condition at noon and frozen immediately and stored at -80°C until further analysis. For gene expression analysis, four weeks old plants growing under moderate conditions were exposed to heat treatment by moving them to 45°C. Leaves of the wild type and the transgenic plants were harvested after 1, 6 and 24 hours and frozen immediately in liquid nitrogen and stored at −80°C until further analysis.

### Construct Preparation and Plant Transformation

The constructs for overexpressing *OsMYB55* were created using the maize ubiquitin promoter. *Agrobacterium*-mediated transformation was used to generate the transgenic plants. The positive transformed plants were selected by the Phosphomannose isomerase (PMI) test [37].

To generate the OsMYB55Pro::GUS construct, a 2139 bp fragment of the *OsMYB55* promoter region was amplified from the genomic DNA by MybproBamHI-F (TGGTGAGGAGGATTGTGCAAGGATCCGCG) and Mybpro-EcoRI-R (CCGGAATTCTTGCACAATCCTCCTCACCA) primers. DNA was isolated from four week old plants grown at the moderate condition using the CTAB method [38]. The amplified fragment was cloned into the molecular cloning site of the pCAMBIA1391Z between the *Bam*HI and *Eco*RI restriction sites to drive the GUS reporter protein. Transgenic rice lines containing the OsMYB55-Promoter were generated using *Agrobacterium*-mediated transformation and the positive lines were selected as previously described by Miki et al.2005 [39].

Constructs containing OsMYB55-Interference RNA (OsMYB55-RNAi) were prepared as described [39]. The 491 bp *OsMYB55* cDNA sequence fragments (with low similarity to other rice genes) were amplified by PCR using MB491F (5′CGTCAAGAACTACTGGAACACC-3′) and MB491R (5′- CCATGTTCGGGAAGTAGCAC-3′) primers. The fragment was cloned into the TOPO pENTER vector (Invitrogen, CA, USA), and the inverted DNA sequences separated by a GUS intron sequence were generated by the site specific recombination method in the pANDA binary vector [39] downstream of the maize ubiquitin promoter using the Gateway LR Clonase Enzyme Mix (Invitrogen, CA, USA). Transgenic rice lines were obtained using *Agrobacterium*-mediated transformation and the positive lines were selected according to [39].

### GUS Histochemical Analysis

Rice plants that carry the *OsMYB55* promoter fused to the GUS reporter gene were used to study the induction and tissue specific expression of the *OsMYB55*. Different plant tissues were harvested one day after exposure to different temperature treatments. Tissues were stained by immersing in 0.1 M sodium citrate-HCl buffer pH 7.0 containing 1 mg/ml 5-bromo-4-chloro-3-indolyl-beta-D-Glucuronide (X-Gluc) (Biosynth, Itasca, IL, USA), followed by vacuum infiltration for five minutes and incubated at 37°C for 16 hours. Chlorophyll was removed by incubating the tissues in 75% ethanol. The samples were stored in glycerol 10% until examination. Freehand, cross-sections were made and observed under a light microscope (Leica DMLS2, Leica, Wetzlar, Germany).

### Microarray Hybridization and Data Analysis

Double-stranded cDNAs was synthesized from 5 µg of total RNA from each sample. Labeled complementary RNA, synthesized from the cDNA was hybridized to the Affymetrix rice whole genome array (Affymetrix Cat. Number: 900601). The hybridization signal of the arrays was obtained by the GeneChip scanner 3000 and quantified by MAS 5.0 (Affymetrix, CA, USA). The probe set 25 measurement was summarized as a value of weighted average of all probes in a set, subtracting the bottom 5% of average intensity of the entire array using a custom algorithm. The overall intensity of all probe sets of each array was further scaled to a target intensity of 100 to enable direct comparison. Data was analyzed using GeneSpring software (Agilent, CA, USA). Genes with 2-fold (or more) change were identified first, and then statistical analysis was done to identify the significant genes within this group (Welch t-test p-value cutoff at 0.05).

### Real-Time RT-PCR Analysis

Quantitative real-time RT-PCR was carried out using specific primers designed from the sequence of the chosen genes. Total RNA was isolated from plant tissues using TRI-Reagent (Sigma-Aldrich, MO, USA). To eliminate any residual genomic DNA, total RNA was treated with RQ1 RNase-free DNase (Promega, WI, USA). cDNA was synthesized from total RNA by using the Reverse Transcription System kit (Quanta, MD, USA). Primer Express 2.0 software (Applied Biosystems, CA, USA) was used to design the primers for the target genes. Relative quantification (RQ) values for each target gene relative to the internal control gene *actin2* were calculated by the 2CΤ method [40].

### Recombinant Protein Production and EMSA

The full length coding regions of OsMYB55 cDNA was amplified by PCR using the following primer pair: Myb55-P28F-BamHI: (5′-GCGGATCCATGGGGCGCGCGCCGT-3′) and Myb55-P28R-HindIII: (5′-CCAAGCTTTGTCAGGGTGTTGCAGAGACCCTGT-3′). The PCR product and the PET15B plasmid (Novagene, WI, USA) were digested with *Bam*HI and *Hind*III. After ligation, the construct was transformed into Arctic Express (DE3) RIL competent cells (Stratagene, CA, USA) according to the manufacturer’s instructions. The recombinant protein of OsMYB55 was purified using the His tag purification Nickel ion system (Qiagen, Hilden, Germany). Electrophoretic Mobility Shift Assay (EMSA) was carried out using the recombinant OsMYB55 protein and the DNA products of the target promoter obtained using the PCR as described in the figures. The Myb binding site TAACTG-box DNA sequence was amplified from the *OsGS1;2, GAD3* and *GAT1* promoters using specific primers. The EMSA assay was carried out using the EMSA kit (Invitrogen, www.Invitrogen.com, Cat # E33075). The DNA/protein complex samples were loaded into a Ready Gel TBE, gradient 4–20% polyacrylamide native gel (Bio-rad Laboratories, www.bio-rad.com) at 200 V for 45 minutes. The DNA in the gel was stained using SYBR® Green provided in the same kit and visualized using the ChemiDoc imaging System (BIO-RAD, Canada).

### Quantitative GUS Activation Analysis

As potential targets for the OsMYB55 transcription factor, DNA sequences corresponding to the *OsGS1;2, GAD3* and *GAT1* promoters were cloned in an intron containing GUS reporter vector, pCAMBIA1391Z. The DNA sequence (1.5–2 kb upstream the ATG start codon of the cDNA) of the different promoters was amplified from the rice genomic DNA using the following primer pairs: OsGS1;2proF: (5′-CACCTGCGGTGAATGGAAGACGTTTG-3′) and OsGS1;2proR: (5′-TGCTCAAAGCAGAAGAGATCTGAATGAG-3′), OsGAD3ProF: (5′-CACCCAGATCAAATGTCAAAAGGGGCG-3′) and OsGAD3ProR: (5′-CTTGCCTGCCGAGCTATCAACC-3′) and OsGAT1ProF: (5′-CACCGACGGAGGAAGTAGTGTGGAACCAT-3′) and OsGAT1ProR: (5′-TGGTGGTAGGGTGCGGC-3′). The resulting fragments were cloned into the TOPO pENTER vector (Invitrogen, CA, USA), and the final construct was made using site specific recombination method into DMC162 gateway vector by LR Clonase Enzyme Mix (Invitrogen, CA, USA). *OsMYB55* was inserted next to the 35S promoter in the DMC32 vector using the forward primer OsMYB55PentF (5′-ATGGGGCGCGCGCCGTG-3′) and the reverse primer OsMYB55PentR (5′-CTATGTCAGGGTGTTGCAGAGACC-3′). This plasmid was used as an activator in the co-transformation transient expression analysis. To normalize the GUS activity values, the firefly (*Photinus pyralis*) luciferase gene driven by the 35S promoter in the pJD312 plasmid (kindly donated by Dr. Virginia Walbot, Stanford University) was used. Equal amounts of DNA from the different plasmid constructs were transformed by particle bombardment into 4-week old tobacco (*Nicotiana plumbaginifolia*) leaves. After incubation for 48 hours at room temperature in the dark, the total protein was extracted from each sample and GUS and luciferase activities were measured. The GUS activity was determined by measuring cleavage of β-glucuronidase substrate 4-methylumbelliferyl β-D-glucuronide (MUG). Luciferase activity was measured using the Luciferase Assay System kit (Cat. E1500) (Promega, www.promega.com) following the manufacturers’ instructions. Empty vectors were used as negative controls in this experiment.

### Amino Acid Quantification

The leaf tissues were freeze dried for 24 h followed by repeated extraction of amino acids for three times using 0.75 mL of 100% methanol. Each extraction was carried out at 70°C for 15 min. The extracts were subjected to chloroform purification by adding 500 µL of extract to 355 µL of water and 835 µL of chloroform. Following centrifugation, the upper phase was collected and freeze dried, then dissolved in deionized water. Total amino acids were assayed [41]. Glutamic acid and arginine were determined in the same extract using L-Glutamic acid and Argenine kits (Megazyme, Bray, Ireland) according to the manufacturer’s instructions.

Proline content was determined according to the protocol previously reported [42]. Briefly, 100 mg of frozen tissues were extracted by 500 µL of 3% sulfosalicylic acid and the supernatant was used for proline quantification. A reaction mixture of 200 µL of glacial acetic acid and 200 µL of acidic ninhydrin was added to 200 µL of extract. The reaction was incubated at 96°C for 60 min and terminated in ice. Proline was extracted from the samples in 1 mL toluene and the absorbance in the upper phase was measured at 520 nm after centrifugation. Proline concentration was determined using a standard curve and calculated on fresh weigh basis.

γ-aminobutyric acid (GABA) was determined in the frozen tissues as described [43]. Briefly, 0.1 g of the frozen tissues was extracted with 400 µL of methanol at 25°C for 10 min. The samples were vacuum-dried, and dissolved in 1 mL of 70 mM lanthanum chloride. The samples were then shaken for 15 min, centrifuged at 13,000 g for 5 min, and 0.8 mL of the supernatant removed to a second 1.5 mL tube. To this solution, 160 µL of 1 M KOH was added, followed by shaking it for 5 min, and centrifugation as mentioned earlier. The resulting supernatant was used in the spectrophotometric GABA determination as follow. The 1 mL assay contained 550**µL of a sample, 150 µL of 4 mM NADP^+^, 200 µL of 0.5 M K^+^ pyrophosphate buffer (prepared by adding 0.15 M phosphoric acid drop-wise to reach the pH 8.6), 50 µL of 2 units GABASE per mL (Sigma-Aldrich, MO, USA) and 50 µL of 20 mM α-ketoglutarate. The initial absorbance was read at 340 nm before adding α-ketoglutarate, and the final absorbance was read after 60 min. The difference in absorbance values was used to prepare a calibration curve. The commercial GABASE enzyme preparation was dissolved in 0.1 M K-Pi buffer (pH 7.2) containing 12.5% glycerol and 5 mM 2-mercaptoethanol. The resulting solution was frozen until use.

### Statistical Analysis

All statistical analyses were performed using SigmaStat (SPSS Inc., Chicago, IL) with an error set at α = 0.05. The significance difference between treatments was tested using Tukey’s Honestly Significant Difference Test.

## Results

### *In-silico* Sequence Analysis

**Figure 1:**
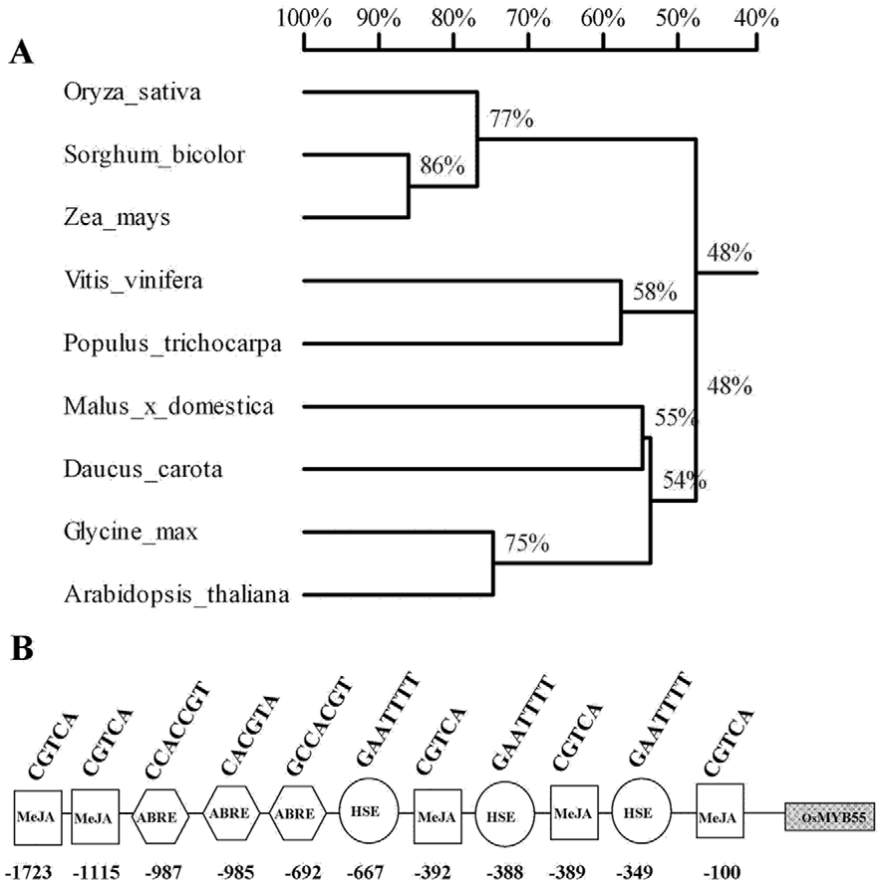
Phylogenetic analysis and cis-acting regulatory elements in the promoter of OsMYB55. Per cent similarity to homologs in other species (**A**), an un-rooted phylogenetic tree was drawn using Tree View based on alignments by CLUSTALX. The amino acid sequences used in this analysis are translated from *Oryza Sativa* (Os05g0553400), *Sorghum bicolor* (242088749), *Zea mays* (226509454), *Vitis vinifera* (296081600), *Populus trichocarpa* (224092242), *Malus x domestica* (71041104), *Dacus carota* (134026414), *Glycine max* (255642827) and *Arabidopsis thaliana* (15242793). Potential cis-elements presented in the *OsMYB55* promoter sequence (**B**), MeJA, cis-acting regulatory element involved in methyl jasmonate -responsiveness; HSE, cis-acting element involved in heat stress responsiveness; ABRE, cis-acting element involved in abscisic acid responsiveness. The numbers below the diagram indicate the position of each cis-element upstream of the ATGG initiation codons.

The 867 bp full length cDNA sequence of *OsMYB55* (Os05g0553400) encodes a R2R3-MYB transcription factor predicted to be 289 amino acids long. This gene was identified during our previous study for nitrogen regulated genes. The BLAST search program was used to identify homologs of *OsMYB55* (http://www.ncbi.nlm.nih.gov/BLAST/). Amino acid sequences of the closest homologs were used to generate a phylogenetic tree using Tree View based on alignments by CLUSTALX (Figure 1a). To understand the regulation of the *OsMYB55* gene, an *in-silico* analysis of the promoter (∼2000 bp) was carried out using PLANT-CARE web sites (http://bioinformatics.psb.ugent.be/webtools/plantcare/html/). The analysis of the *OsMYB55* promoter revealed the presence of several *cis*-acting regulatory elements (CAREs) and transcription factor binding sites (TFBS) related to different physiological responses. The major cis-elements in the promoter are shown in Fig. 1b and contain three ABA responsive elements (ARE), five methyl jasmonate responsive elements (MeJA-R) and three cis-acting elements involved in heat stress responsiveness (HSE). Additionally, the *OsMYB55* promoter has TFBS for many transcription factors already identified such as DOF, MYB, AP2 and WRKY.

### Induction of *OsMYB55* by High Temperature

**Figure 2:**
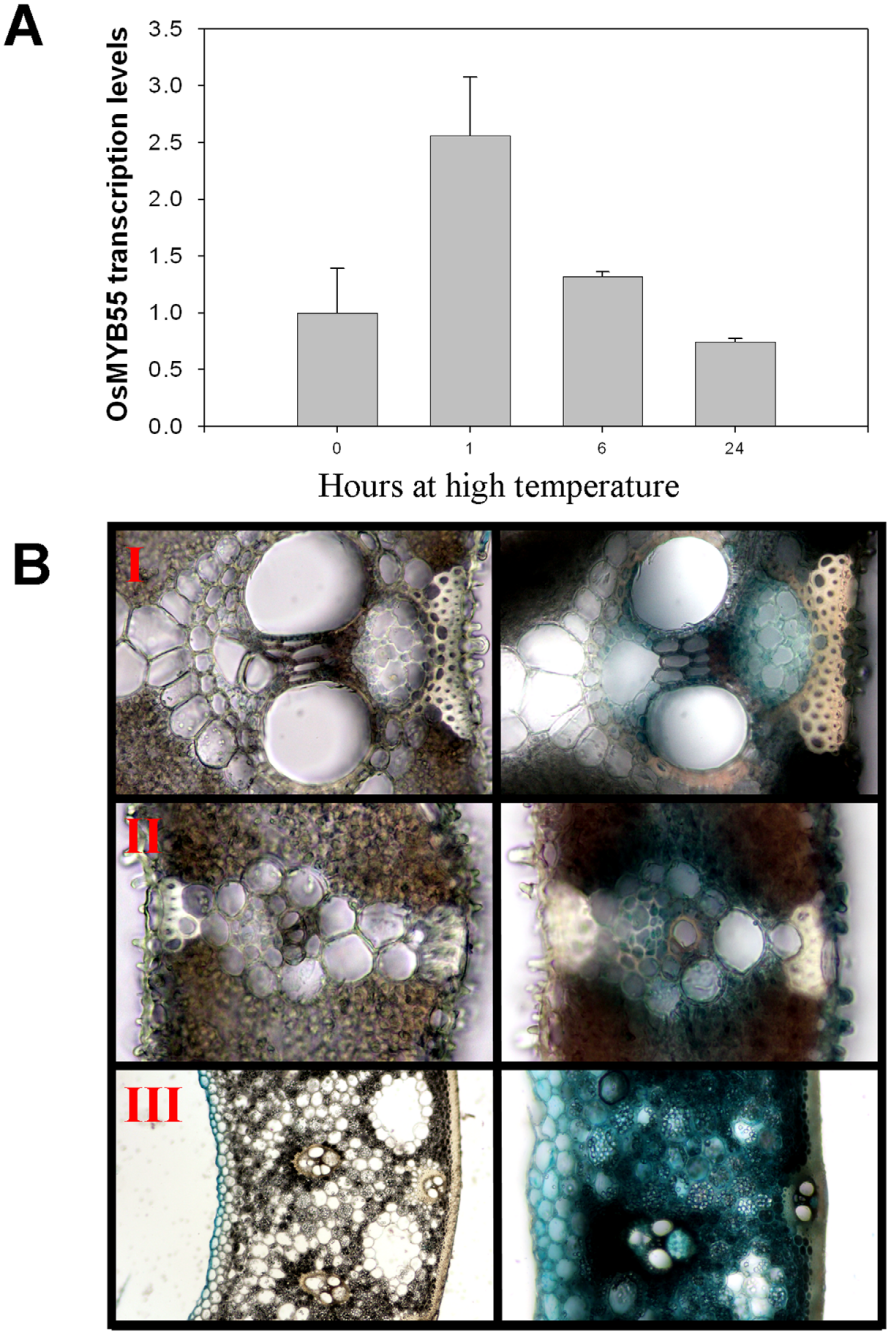
Upregulation of the OsMYB55 expression by high temperature. Quantitative transcript levels of *OsMYB55* in the leaves of four weeks old rice plants exposed to 45°C as a high temperature for 0, 1, 6 and 24 hr (**A**). Data are means ± SD (n = 3). Histochemical staining of the GUS protein in rice plants carrying the GUS reported gene under control of *OsMYB55* promoter (**B**). Plants were exposed to 45°C and samples were collected after treatment. Cross-sections of the leaf med vein (I & II) and leaf blade (III) were prepared and visualized under the light microscope. The GUS stain was visible as early as one hour after treatment.

To study the response of native *OsMYB55* to high temperature, wild type plants were exposed to 45°C for 24 hours and leaves were sampled. Quantitative real-time RT-PCR analysis showed *OsMYB55* expression was induced in wild type plants after one hour from the exposure to high temperature and declined to the basal level after 6 and 24 hours (Figure 2a). This induction also was observed in the transgenic plants that carry the *OsMYB55* promoter fused to the reporter GUS gene. However, induction was best observed 24 hours after heat treatment at 45°C (Figure 2b).

### *OsMYB55* is more Specifically Expressed at the Vegetative Stage

**Figure 3:**
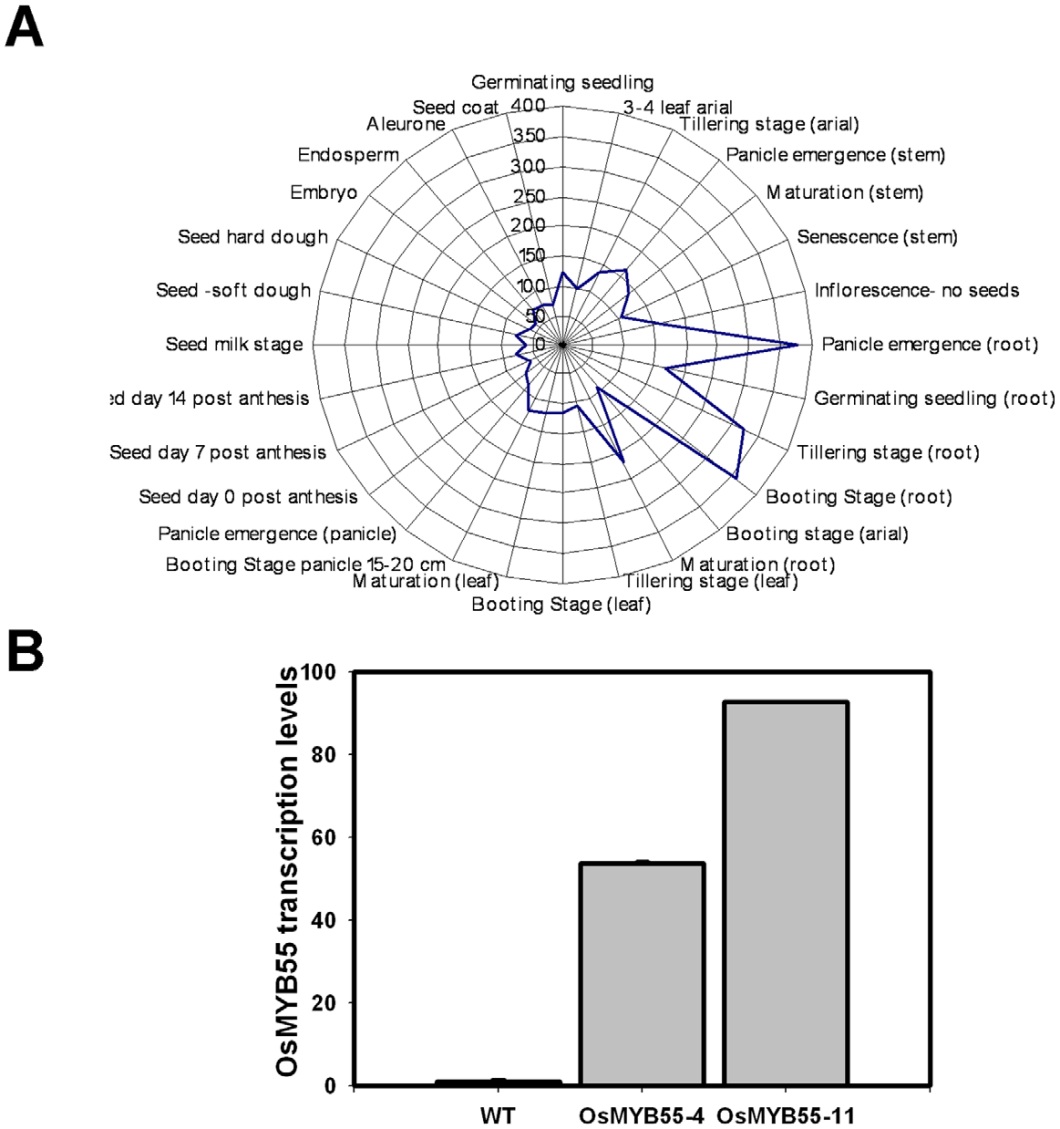
Quantitative relative gene expression of *OsMYB55.* In the wild-type rice plant organs throughout the life cycle (**A**) and in the leaves of the rice *OsMYB55* overexpression lines (**B**).

Expression analysis of the *OsMYB55* revealed that transcript levels are higher at the vegetative stages up to tillering and the inflorescence stage. The transcription was higher in the root tissues compared to the leaves at these stages. However, its lowest expression level was observed in all seed development stages and at seed maturation (Figure 3a). To study the role of *OsMYB55* in plants, transgenic *Oryza sativa* cv. Kaybonnet plants that overexpressed the full length cDNA of *OsMYB55* were generated. Expression analysis of the transgenic plants showed that *OsMYB55* transcript levels were between fifty and ninety times higher in the transgenic lines than in the wild type plants (Figure 3b).

### Overexpression of the *OsMYB55* Improves Plant Growth under High Temperature

**Figure 4:**
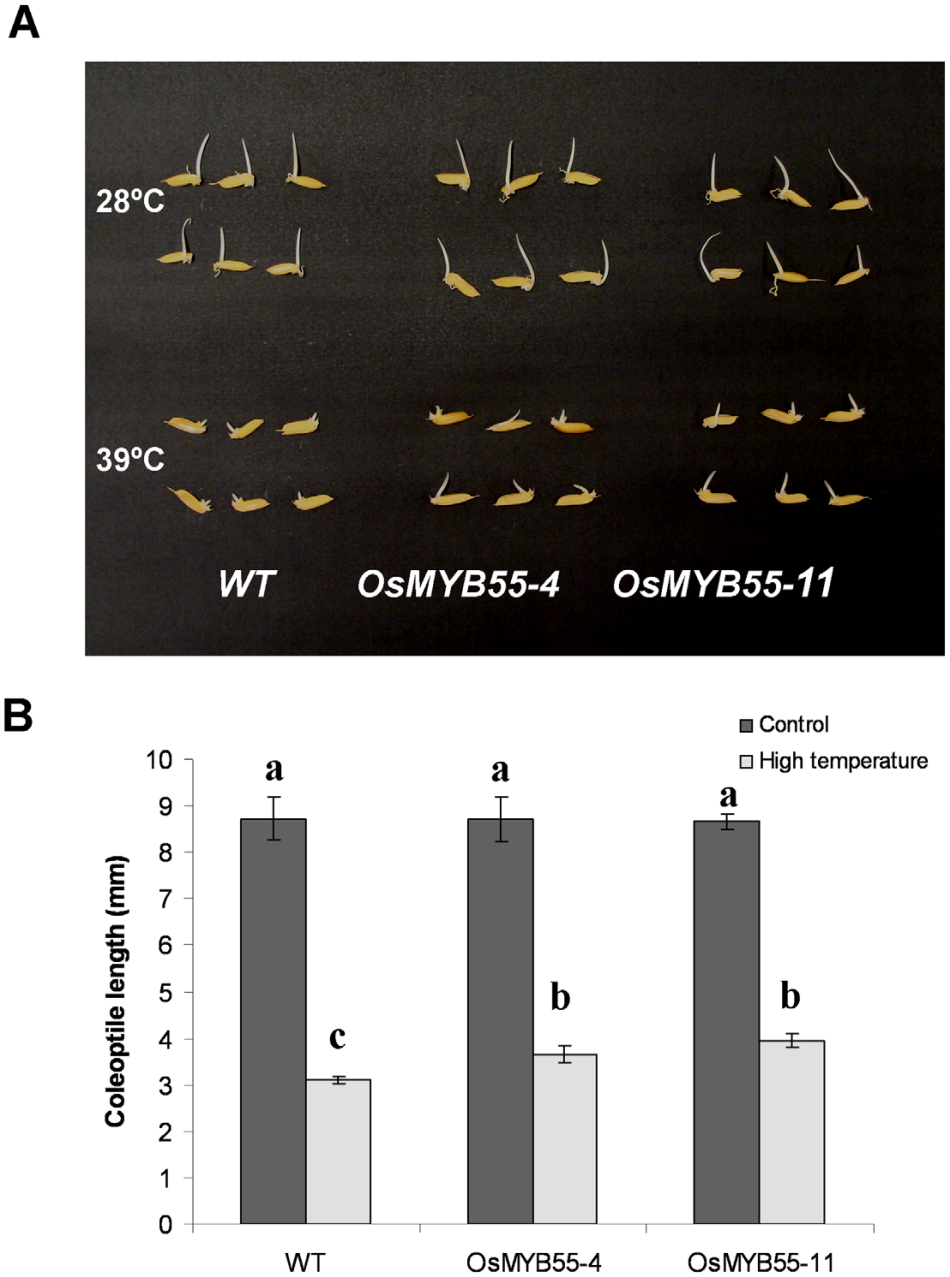
High temperature effects on coleoptile growth in wild-type and *OsMYB55* overexpression plants. WT (Oryza sativa cv. Kaybonnet) and *OsMYB55* overexpression lines (OsMYB55-4 and OsMYB55-11) seeds were germinated and grown either at 28°C or 39°C for five days (**A**), and coleoptile lengths (**B**). Data are means ± SD (n = 3). Each replicate consisted of 25 seedlings. Starred high temperature bars indicate significant difference from WT grown at high temperature.

Since *OsMYB55* is induced during exposure to high temperatures, we investigated whether it has a role in plant tolerance to high temperature. Experiments were carried out at different developmental stages to check the effect of *OsMYB55* overexpression on plant thermotolerance. At very early seedling stages, coleoptiles of plants that overexpressed *OsMYB55* grew longer than the wild type at 39°C for 5 days (Figure 4a,b).

**Figure 5:**
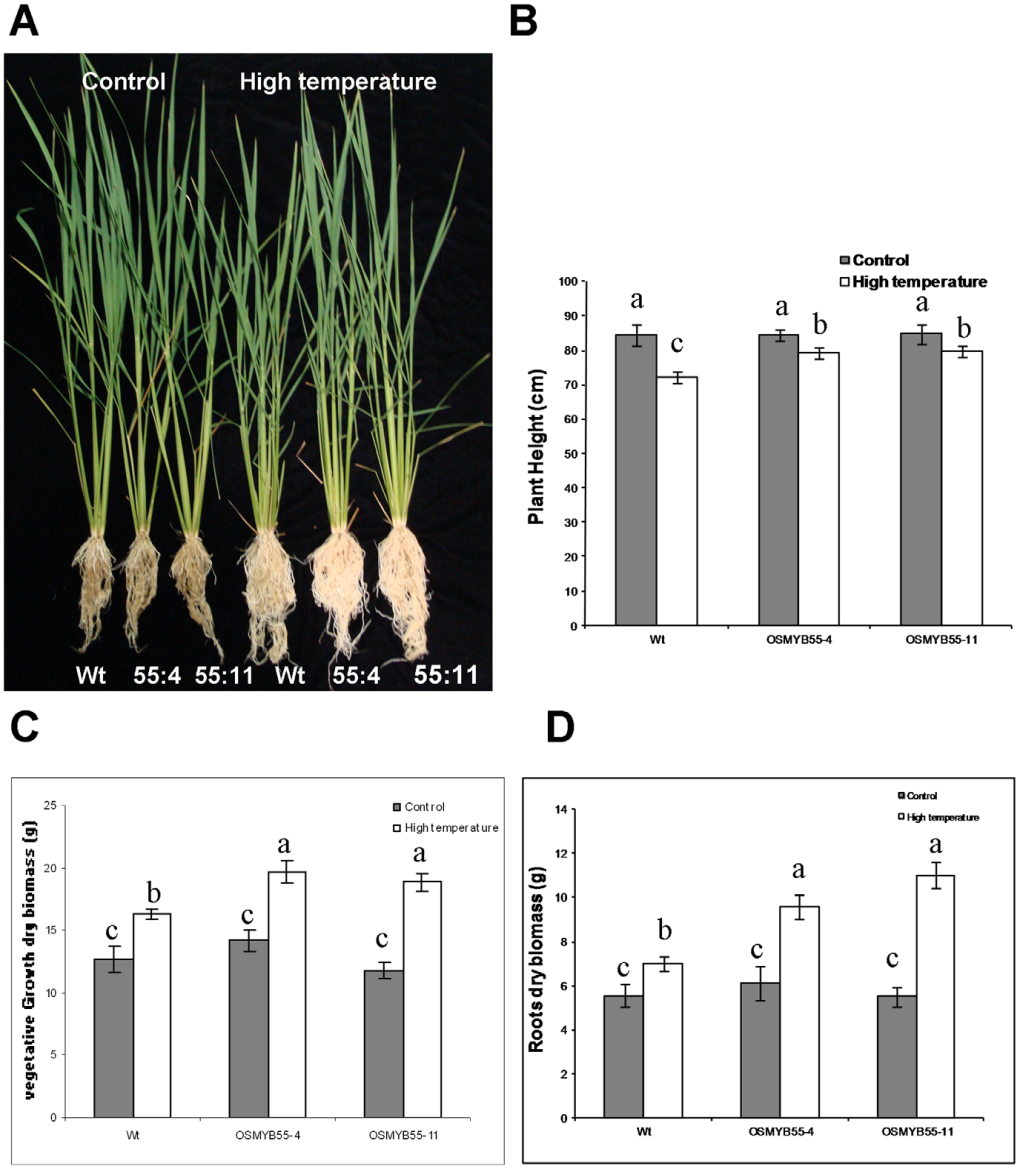
High temperature effects on plant growth under long day condition. Plants were grown in Turface either at 29°C (Control) or at 35°C (high temperature) for four weeks (**A**), plant height (**B**), shoot biomass (**C**) and root biomass (**D**) were measured at harvest. Data are means ± SD (n = 6).

**Figure 6:**
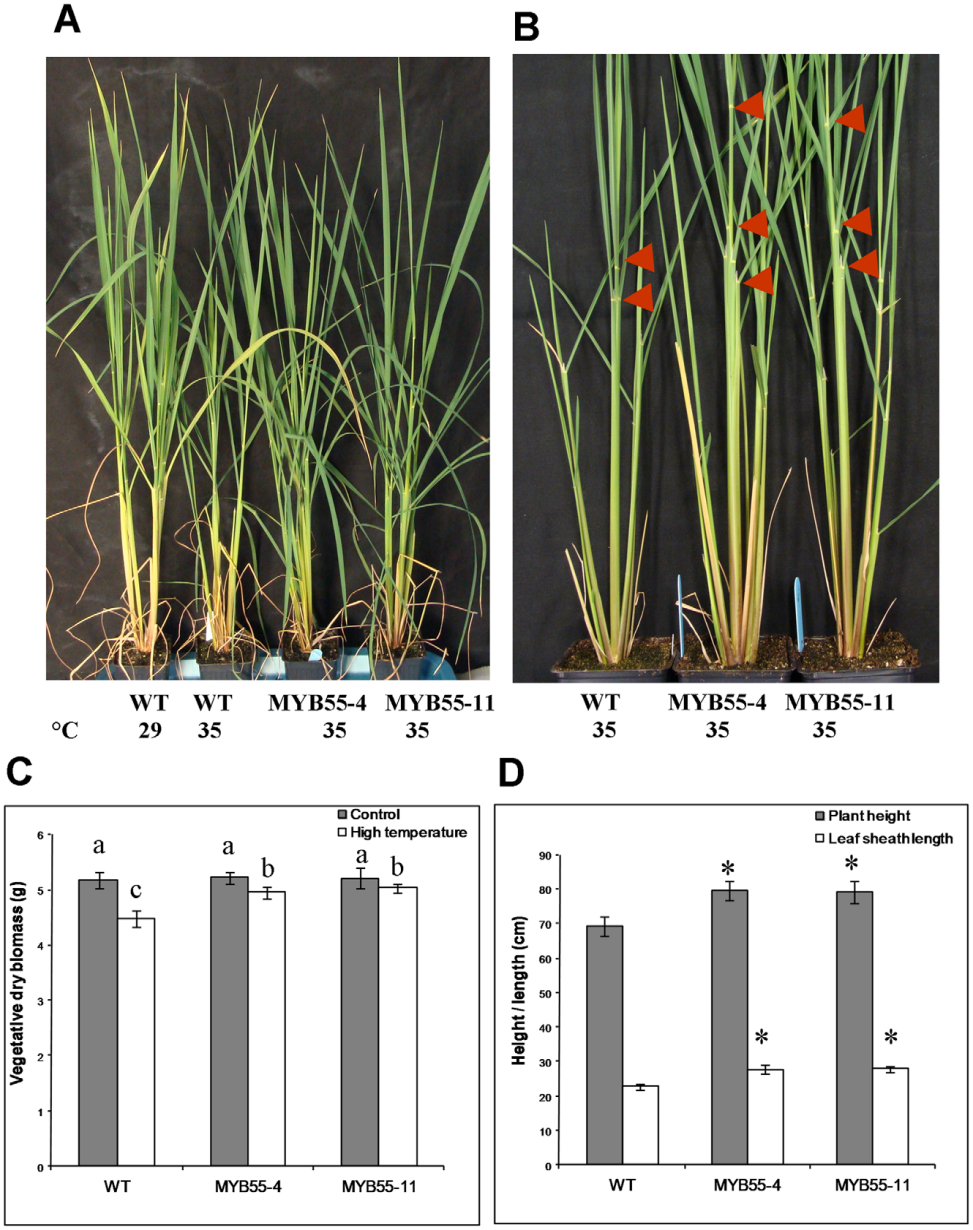
High temperature effects on plant growth under neutral day condition. Plants were grown in Peat-moss:vermiculite (1∶4) mix either at 29°C (Control) or at 35°C (high temperature) for four weeks (**A & B**) no significant difference was observed between the wild type and OsMYB55 overespression plants grown under moderate temperatures. Significant differences of shoot dry biomass (**C**), and plant height and leaf sheath length (**D**) between wild type and OsMYB55 overexpression plants. Data are means ± SD (n = 6).

To further test if the *OsMYB55* overexpression can confer a thermotolerant phenotype to the plants growing at constant high temperature, plants were grown in Turface to facilitate root cleaning in order to monitor root growth. Shortly after germination, plants were moved to high temperatures for four weeks. Under moderate temperature, there was no significant difference between shoot and root growth of the wild type and the transgenic plants (Figure 5a, b, c, d). Wild type plants grown under high temperature showed a decrease in plant height and an increase in dry biomass. The transgenic plants grown in the same conditions showed less reduction in plant height and a significant increase in plant biomass and root biomass compared to the wild type (Figure 5a, b, c, d). Rice varieties with different day length requirements grow under different culture condition around the world. In order to test the effect of *OsMYB55* over-expression on imparting tolerance to high temperature under different culture conditions, the same experiments were repeated using regular soil consisting of 4∶1 volumes of Vermiculite: Peat-moss and grown under 12 hr light (∼500 µmol m^−2^s^−1^) at 35°C and 12 hr dark at 26°C or under 29°C and 23°C day/night temperatures. Results confirmed the phenotypic data obtained from the long day experiments and showed that growing the plants at high temperature for four weeks reduced plant height and decreased plant dry biomass in both wild type and transgenic plants. However, it was apparent that *OsMYB55* overexpression moderated the negative effects of high temperature resulting in greater plant dry biomass, plant height and leaf sheath length than wild type plants (Figure 6a, b, c, d).

Plants grown under a continuous high temperature caused deformations of the inflorescences and resulted in a complete seed set failure (Figure S1) compared to the plants grown under moderate temperature. This effect was more severe in the plants grown under long day conditions than neutral day conditions, for both wild type and transgenic plants.

**Figure 7:**
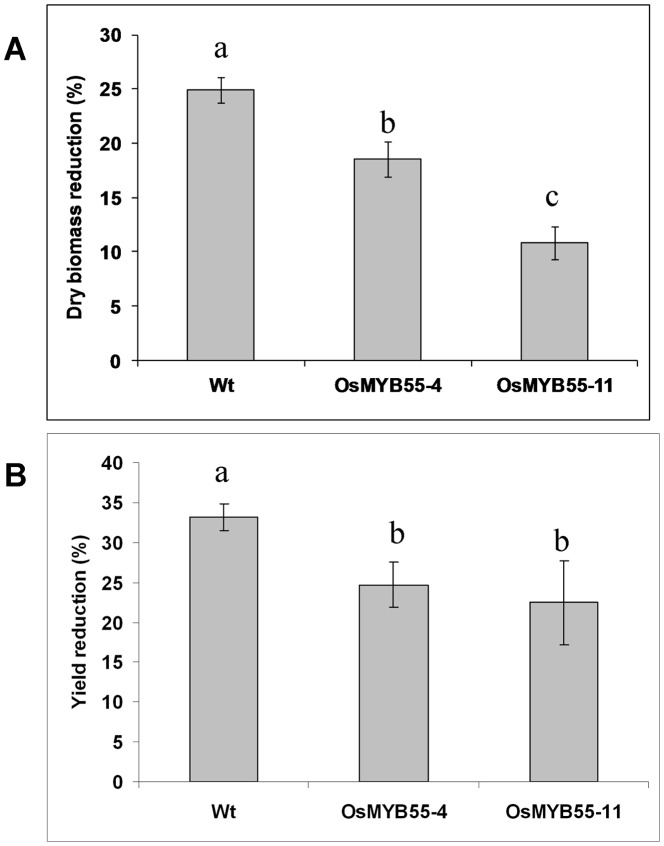
Reduction of dry biomass and grain yield in rice wild type and OsMYB55 overexpression lines in response to high temperature. Plants were grown either at moderate temperature or at high temperature under neutral day condition for four weeks and moved to moderate temperature until harvest. The reduction of the dry biomass (**A**) and grain yield (**B**) in the plants grown at high temperature were calculated relative to the plants grown at optimum temperature.

Further testing of the *OsMYB55* overexpression rice plants was conducted by transferring the plants after the exposure either to moderate or high temperature to complete their life cycle under the moderate condition. This treatment caused a significant reduction in both total dry biomass and grain yield in both wild type and transgenic plants. However, this reduction was less in the transgenic lines (Figure 7a, b). Although, *OsMYB55* overexpression failed to enhance plant thermotolerance during inflorescence and seed set under continuous high temperature, it moderated the reduction in the grain yield caused by high temperatures when the plants were grown at high temperatures for four weeks followed by moderate temperatures for the rest of the life cycle (Figure 7a, b). It is obvious that growing rice plants for the first five weeks of their life cycle under high temperature negatively affected the grain yield at harvest, but this reduction was less significant in the overexpression plants.

Finally, we generated RNAi lines for OSMYB55 which had up to a 50% decrease in expression of this gene. However, under the same conditions tested for the over-expression lines we saw no change in the heat tolerance in these lines. This is likely due to either functional redundancy or to requiring various threshold levels of expression changes to see this phenotypic change.

### *OsMYB55* Over-expression Leads to an Increase in Total Amino Acid Content, Especially under High Temperature

**Figure 8:**
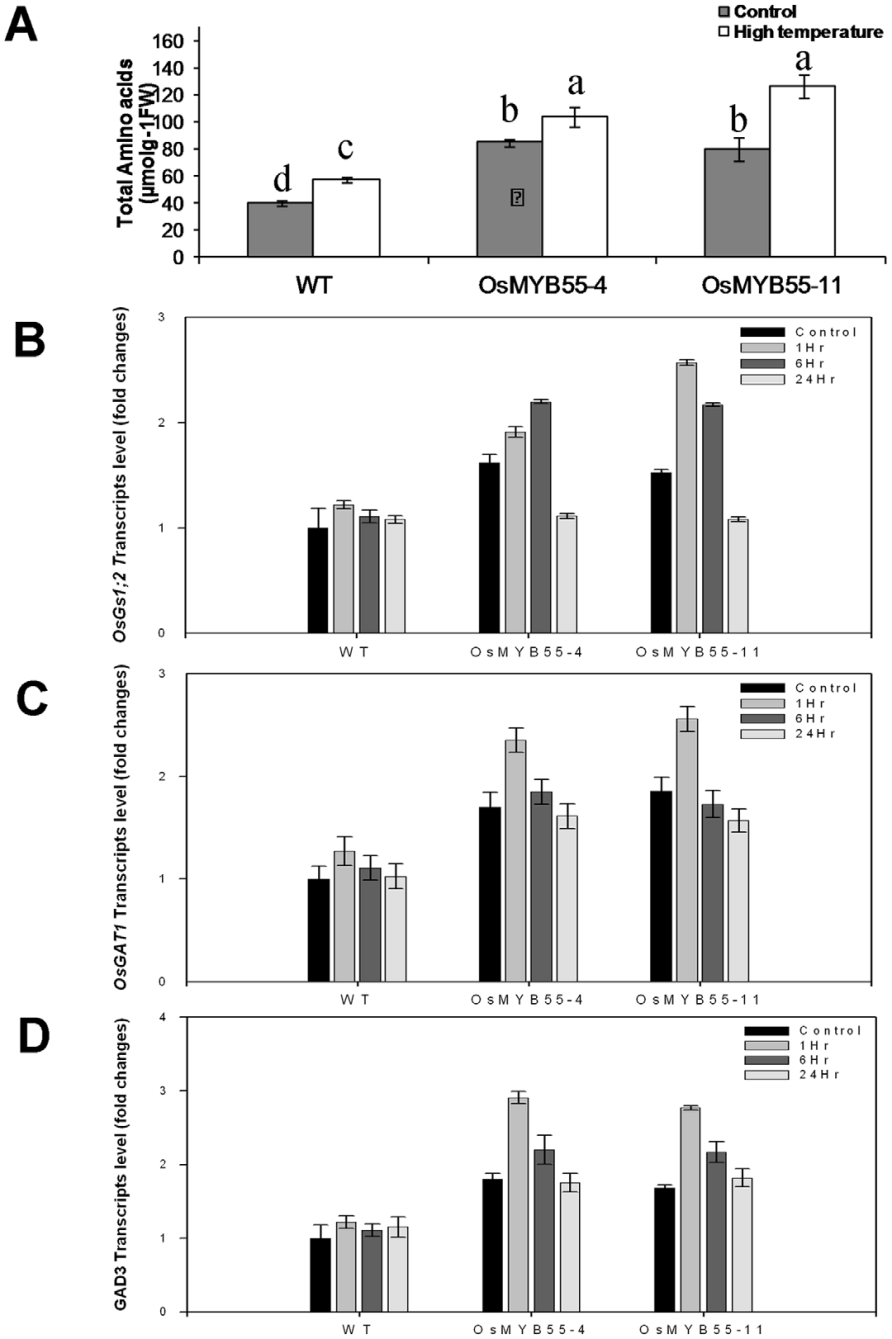
High temperature influences amino acid biosynthesis in rice plants. Leaf total amino acids content of wild type and transgenic plants grown under long day condition and at either moderate or high temperature for four weeks (**A**). Quantitative relative gene expression of OsGs1;2, GAT1 and GAD3 in the leaves of plants grown either at moderate temperature (control) or exposed to 1, 6 and 25 hr to high temperature (45°C) (**B–D**). Data are means ± SD (n = 3) and are representative of similar results from three independent experiments.

To understand the physiological and molecular mechanisms underlying the enhancement of plant thermotolerance by *OsMYB55*, tissues were collected for various analyses. Previous published studies indicated that plant thermotolerance is a complicated multi-factorial trait. Biochemical analysis was carried out on sugars, starch, hydrogen peroxide and various nitrogenous components to determine whether any of these showed significant differences between wild-type and overexpression lines. Of these, the leaves of the transgenic plants overexpressing *OsMYB55* had higher total amino acid content under moderate condition compared to the wild type plants (Figure 8a). Exposing the wild type plants to high temperature caused a modest increase in leaf amino acid content while the transgenic plants were able to maintain a higher level of total amino acids during growth under high temperature.

### OsMYB55 Alters the Expression of Genes Involved in Amino Acid Metabolism

Based on the total amino acid analysis results, we suspected that OsMYB55 might have a role in the activation of some genes involved with amino acid metabolism. To test this hypothesis, a genome-wide transcriptome analysis was conducted using microarray analysis of the transgenic and wild type plants exposed to high temperatures. Two candidate genes important for amino acid production were found to be up-regulated, namely class I glutamine amidotransferase (*GAT1*, accession number: BAD08105.1) and glutamate decarboxylase 3 (*GAD3*, accession number: AY187941.1). GAT1, also known as carbamoyl phosphate synthetase is inovlved in the first committed step in arginine biosynthesis in prokaryotes and eukaryotes [44]. The *GAD* genes are involved in converting the L-glutamic acid into GABA [45]. Quantitative real time PCR revealed the up-regulation of those two genes one hour after the exposure of rice to high temperature (45°C), although their transcript level decreased to the basal level after 24 hours (Figure 8b,c). The microarray analysis can miss more subtle changes in gene expression due to the statistical analysis used. Therefore, real time PCR was performed using primers from other genes, involved in amino acid biosynthesis and transport. Beside the above mentioned *GAT1* and *GAD3*, another gene, glutamine synthetase (*OsGS1;2*, accession number: AB180688.1*)* was found to be up regulated in the leaf of the transgenic plants one hour after the exposure to high temperature (Figure 8d). The *OsGS1;2* is involved in converting the glutamine into glutamic acid and represents one of the early steps in N metabolism and amino acids biosynthesis. Further analysis with the microarray data was done by AgriGO – the tools developed by Zhen Su’s laboratory in the China Agriculture University [46] using the lists either only up-regulated in the Myb transgenic plants (451 genes, Figure S2) or in the wild type plants (307 genes, Figure S2) during heat treatment. No GO terms were enriched in either case. We then analyzed the upregulated gene lists in Myb transgenic plants (451+2427 genes, Figure S2) or in the wild type plants (307+2427 genes, Figure S2), and found that protein localization (GO:0008104), protein transport (GO:0015031) were enenriched in the Myb plants but not in the wild type plants (Table S1).

### OsMYB55 Binds to and Activates Different Targets Involved in Amino Acid Metabolism

**Figure 9:**
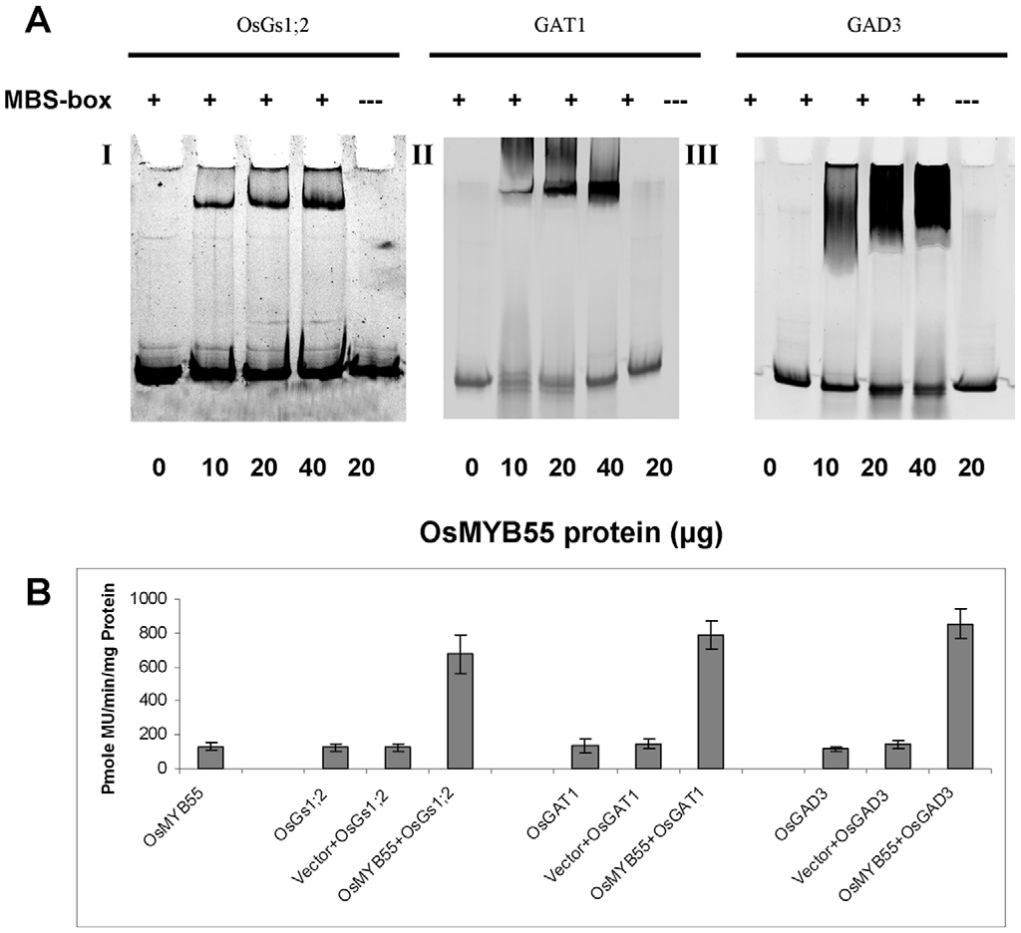
Binding and the activation of the OsGS1;2, GAT1 and GAD3 promoters by OsMYB55 Electrophoretic Mobility Shift Assay (EMSA). Binding of different concentrations of OsMYB55 protein to 200 ng of DNA containing one copy of the MB-box (**A**). Transcription activation was carried out by using GUS based transient assay and 4-methylumbelliferyl b-Dglucuronide (MUG) as the substrate (**B**). The 4-methyl umbelliferone (MU) was used to generate the standard curve. Bars represent mean ± SE. (n = 6).

To investigate the binding and the activation of the identified genes (*OsGS1;2, GAT1* and *GAD3*) by OsMYB55, DNA sequences corresponding to the promoters of the target genes was analyzed using PLANT-CARE web sites (http://bioinformatics.psb.ugent.be/webtools/plantcare/html/). The results showed that all three promoters have a potential binding site for the MYB protein. This CAGTTA cis-element motif is located at 1079 bp, 460 bp and 554 bp from the first ATG codon of the *OsGS1;2, GAT1* and *GAD3* cDNA, respectively. To check the possibility that OsMYB55 protein binds to the CAGTTA-box *in vitro*, recombinant OsMYB55 protein was produced in *E. coli* and used for Electrophoretic Mobility Shift Assays (EMSA). The results demonstrated that OsMYB55 strongly binds to the *OsGS1;2, GAT1* and *GAD3* promoter sequences containing the CAGTTA box motif (Figure 9a).

Binding of the OsMYB55 protein to the promoter of the *OsGS1;2, GAT1* and *GAD3* supports the idea that OsMYB55 enhances amino acid content by activation of these genes. To investigate this hypothesis, a transcription activation assay using a transient gene expression strategy was carried out using β-glucorinidase (GUS) as a reporter protein. The results demonstrate that OsMYB55 activated the expression of *OsGS1;2, GAT1* and *GAD3* in tobacco epidermal cells by almost 8-fold compared to the control experiment (Figure 9b). Together, these results indicatde that OsMYB55 directly regulates the expression of *OsGS1;2, GAT1* and *GAD3*.

### *OsMYB55* Overexpression Leads to Increase in Glutamic Acid, GABA, Arginine and Proline Particularly under Heat Stress

**Figure 10:**
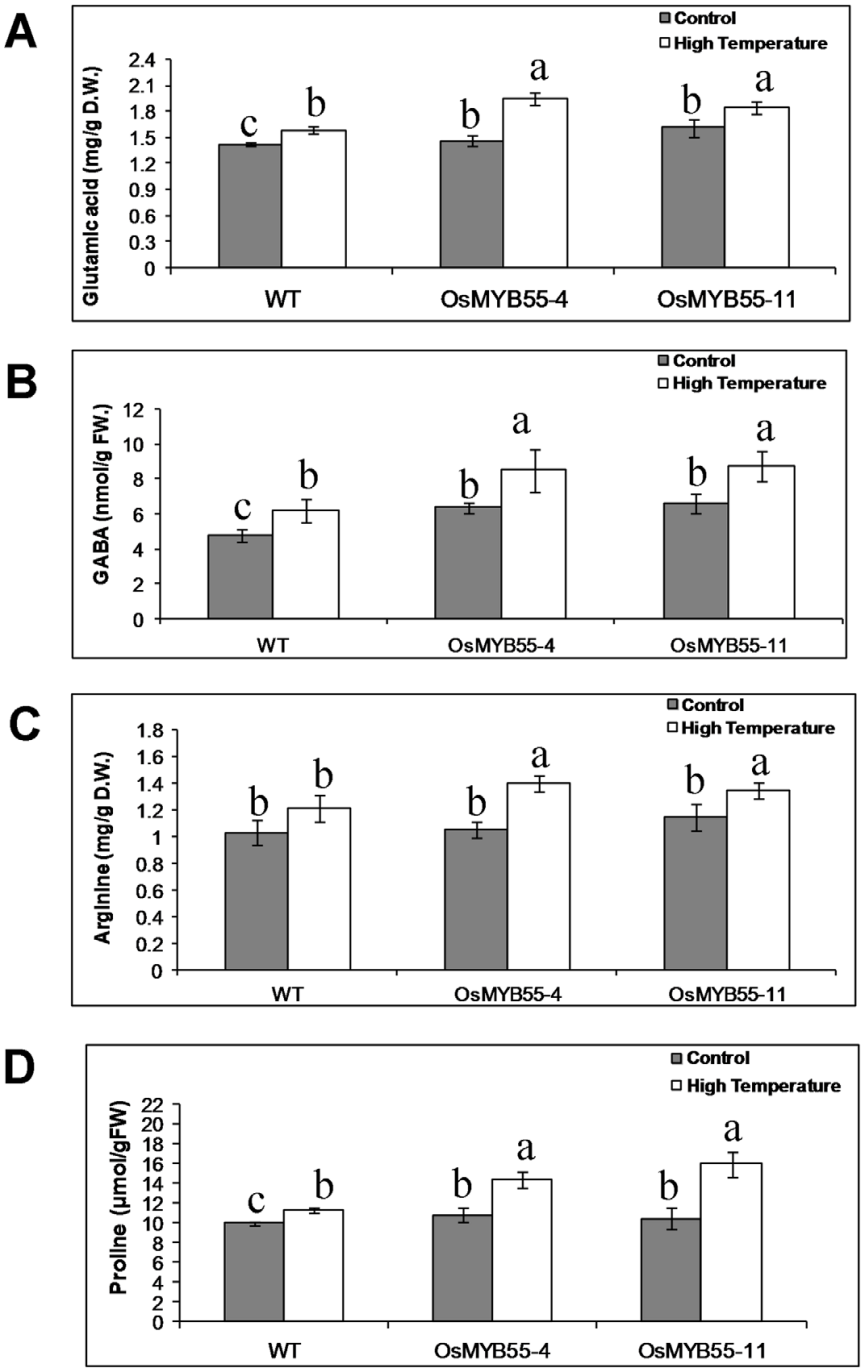
Amino acid content of Rice leaves as affected by high temperature. Leaf content of glutamic acid (**A**), GABA (**B**), arginine (**C**) and proline (**D**) in the wild type and OsMYB55 overespression plants growing under long day condition. Gray bars represent the plants grown at 29°C and white bars present the plants grown at high temperature (35°C), Bars represent mean ± SD. (n = 4).

Glutamic acid is one of the first amino acids to be synthesized from nitrogen compounds and can be converted into other amino acids. Consistent with the *OsGS1;2* transcript level, transgenic plants that overexpressed *OsMYB55* had similar leaf glutamic acid content under moderate conditions (Figure 10a). In response to the high temperature, glutamic acid content increased in the leaves of both wild type and transgenic lines. However, this increase was significantly higher in the transgenic lines compared to the wild type plants (Figure 10a).

Gamma aminobutyric acid (GABA) is induced under different stress condition and is believed to have a role in plant tolerance to stress [47]. In this study, under moderate conditions, we found an increase in the leaf GABA content of the transgenic lines overexpressed *OsMYB55* compared to the wild type plants. Upon the plant exposure to high temperatures, a significant increase in leaf GABA content was observed in both wild type and transgenic plant. However, this increase was more obvious in the transgenic plants (Figure 10b).

Arginine is required for polyamine biosynthesis in plants which have been reported to be involved in several plant developmental and stress conditions including high temperature [48], [49]. Our results show that transgenic plants overexpressing *OsMYB55* had the same level of arginine as wild type plants when growing under moderate temperature for four weeks (Figure 10c). In contrast, growing the plants under high temperature resulted in an increase in arginine content in the leaves of the wild type and transgenic plants. However, this increase was significantly higher in the transgenic plants (Figure 10c).

Several studies pointed to the importance of proline in plant tolerance to high temperature. No significant difference was found in proline content between the wild type and transgenic plants under moderate condition (Figure 10d). High temperature increased leaf proline content in wild type plants, while a much greater increase was exhibited in the transgenic plants.

## Discussion

In the present study we identified a MYB transcription factor that enhances rice plant tolerance to high temperature during vegetative growth. The response of plants to heat stress is complex. Heat shock proteins are the main components in this response, although other factors have been suggested to have important roles in plant adaptation to high temperature. The function of heat shock proteins is to act as molecular chaperones and to resolubilize protein aggregation caused by heat stress [11]. Plant survival under high temperature conditions requires maintenance of homeostasis not only with heat shock proteins but other stress reducing components. Several studies have suggested the importance of other pathways in conferring heat tolerance in plants which may involve hormones, antioxidants, osmosolutes and carbohydrates [50]. However, little is known about the involvement of the large family of MYB transcription factors in plant tolerance to high temperature. Here, we found that *OsMYB55* is induced by high temperature with similar results having been reported for the *Arabidopsis MYB68* gene [36] which has a poor homology with the rice *OsMYB55*. In the tree analysis, we found orthologous genes in other species. However, currently there is no evidence about the involvement of any of these genes in heat tolerance, and it would be interesting to test these to see if they play the same role as OsMYB55 in the other species. Overexpression of *OsMYB55* improved plant growth and productivity under high temperature conditions. The transgenic plants maintain higher plant height and more dry-biomass compared to the wild type plants grown under high temperature. Exposure of both wild type and *OsMYB55* overexpression plants for four weeks in the beginning of the life cycle to high temperature under a neutral daylight condition, decreased grain yield at harvest. However, this reduction was significantly less in the transgenic plants. Growing the plants under a high temperature and long day condition for four weeks increased dry biomass. However, the transgenic plants had a higher dry biomass compared to the wild type plants. Together, these results indicate that the transgenic lines grow and perform better under high temperature than wild-type. Growing the rice plants for four weeks at high temperature decreased grain yield and this is likely due to the effect of high temperature on flower initiation which occurs during the vegetative stage. To explore the function of the *OsMYB55* in enhancing plant growth under high temperature, different biochemical and molecular analyses were performed. Several amino acids and nitrogenous compounds play a vital role in plant tolerance to different stress conditions [51]. In the present study, plants that overexpressed *OsMYB55* had an increase in total amino acid content. Transcriptome analysis did result in the identification of three potential targets for the rice OsMYB55, namely *OsGS1;2, GAT1* and *GAD3*. OsMYB55 binds *in vitro* to the promoters of these genes and transactivates them in tobacco leaf cells.

*OsMYB55* overexpression leads to improved heat tolerance and enhances the level of total amino acids and glutamine acid, proline, arginine, and GABA in particular. It has been reported that the enhancement of amino acid biosynthesis, especially of proline, GABA and arginine has a significant impact in improving plant tolerance to different stress conditions [47]. These amino acids have multifunctional roles in plants that help plant tissues resist and recover from stress [52]. Additionally, arginine has been reported to have a major role in improving wheat yield under high temperature conditions [16]. Furthermore, increasing the amino acid content could have an impact in activating downstream components leading to enhancing plant heat tolerance. For instance, arginine is the precursor for polyamine biosynthesis and has been shown to be involved in plant tolerance to different stresses [53], [54]. It should be noted that while proline content was increased in the overexpression lines in response to high temperature, there were no significant difference in the expression of the genes involved in proline biosynthesis. These results suggest that the increase in proline content is indirect and could be due to other pathways including protein breakdown [55]. Finally, we do not exclude other mechanisms that could be involved in enhancing the tolerance of these plants to high temperatures other than the changes of amino acid levels. As the microarray analysis results showed that some biological processes including protein localization (GO:0008104) and protein transport (GO:0015031) are upregulated in the Myb transgenic plants but not in the wild type plants (Table S1), further investigation would be required to demonstrate how this *Myb* gene is involved in this regulation.

In conclusion, overexpression of *OsMYB55* leads to increased heat tolerance of rice plants during the vegetative stage. This leads to increased biomass and if the plants are subsequently grown under moderate conditions leads to increased seed yield. One explanation for this is that increasing the amino acid content at the vegetative stage through the action of *OsMYB55* could help the plant in the flower initiation process which occurs at this stage thus resulting in a higher yield when compared to the wild type plants. This trait will become of increasing importance as crop yields in many important rice growing regions are decreased due to higher temperatures given global warming. Therefore, it is of great importance to explore different crop genetic solutions to ameliorate this problem. Modulating the expression of *OsMYB55* either by itself or in combination with other genes may be one potential solution.

## Supporting Information

Figure S1 Rice panicles as affected by high temperature. High temperature inhibits panicle length (**A**). High temperature caused panicle and floret deformation at flowering stage when plants were grown under long day (**C**) or neutral day (**D**) compared to the panicle of rice plants grown at moderate temperature (**B**). Failure of grain filing at harvest stage in the plants grown at 35°C under long day (**F**) or neutral day (**G**) compared to the plants grown at moderate temperature (**E**).
(PDF)

Figure S2 Transcriptome changes between the wild type and transgenic plants upon the exposure to high temperature. The Venn diagrams represent the number of genes that were found to be significantly differentially expressed between OsMYB55 overexpression plants and wild-type (WT) in response to high temperature treatment. Four weeks old rice plants were exposed to high temperature (45°C) for one hour. The upper Venn diagram (**A**) represents the number of genes that were found to be up-regulated in the transgenic and wild plants in response to high temperature, and the lower Venn diagram (**B**) shows the number of genes that were found to be down-regulated. Genes in the medium are regulated in both transgenic and wild type plants.
(PDF)

Table S1 Significantly enriched upregulated biological processes in the wild type and Myb transgenic plants during heat treatment
(PDF)

## References

[1] IPCC (2007) Summary for policy makers. In: Climate change. The physical Science Basis. 9: 1–18.

[2] Ashraf M, Saeed MM, Qureshi MJ (1994) Tolerance to high temperature in cotton (*Gossypium hirsutum* L.) at initial growth stages. Environ Exp Bot 34: 275–283.

[3] Kolupaev Y, Akinina GE, Mokrousov AV (2005) Induction of heat tolerance in wheat coleoptiles by calcium ions and its relation to oxidative stress. Russian J Plant Physiol 52: 199–204.

[4] Endo M, Tsuchiya T, Hamada K, Kawamura S, Yano K, et al (2009) High temperatures cause male sterility in rice plants with transcriptional alterations during pollen development. Plant Cell Physiol 50: 1911–1922. 19808807 10.1093/pcp/pcp135

[5] Jagadish SV, Muthurajan R, Oane R, Wheeler TR, Heuer S, et al (2010) Physiological and proteomic approaches to address heat tolerance during anthesis in rice (*Oryza sativa* L.). J Exp Bot 61: 143–156. 19858118 10.1093/jxb/erp289 PMC2791117

[6] Morita S, Yonemaru J, Takanashi J (2005) Grain growth and endosperm cell size under high night temperatures in rice (*Oryza sativa* L.). Ann Bot 95: 695–701. 15655104 10.1093/aob/mci071 PMC4246861

[7] Lin CJ, Li CY, Lin SK, Yang FH, Huang JJ, et al (2010) Influence of high temperature during grain filling on the accumulation of storage proteins and grain quality in rice (*Oryza sativa* L.). J Agric Food Chem 58: 10545–10552. 20839801 10.1021/jf101575j

[8] Lin-Wang K, Micheletti D, Palmer J, Volz R, Lozano L, et al (2011) High temperature reduces apple fruit colour via modulation of the anthocyanin regulatory complex. Plant Cell Environ 43: 1176–1190. 10.1111/j.1365-3040.2011.02316.x 21410713

[9] Peng S, Huang J, Sheehy JE, Laza RC, Visperas RM, et al (2004) Rice yields decline with higher night temperature from global warming. Proc Natl Acad Sci U S A 101: 9971–9975. 15226500 10.1073/pnas.0403720101 PMC454199

[10] Maestri E, Klueva N, Perrotta C, Gulli M, Nguyen HT, et al (2002) Molecular genetics of heat tolerance and heat shock proteins in cereals. Plant Mol Biol 48: 667–681. 11999842 10.1023/a:1014826730024

[11] Lee U, Rioflorido I, Hong SW, Larkindale J, Waters ER, et al (2007) The Arabidopsis ClpB/Hsp100 family of proteins: chaperones for stress and chloroplast development. Plant J 49: 115–127. 17144892 10.1111/j.1365-313X.2006.02940.x

[12] Baniwal SK, Bharti K, Chan KY, Fauth M, Ganguli A, et al (2004) Heat stress response in plants: a complex game with chaperones and more than twenty heat stress transcription factors. J Biosci 29: 471–487. 15625403 10.1007/BF02712120

[13] Kotak S, Larkindale J, Lee U, Koskull-Doring P, Vierling E, et al (2007) Complexity of the heat stress response in plants. Curr Opin Plant Biol 10: 310–316. 17482504 10.1016/j.pbi.2007.04.011

[14] Singh A, Grover A (2008) Genetic engineering for heat tolerance in plants. Physiol Mol Biol Plants 14: 155–166. 23572882 10.1007/s12298-008-0014-2 PMC3550655

[15] Ogweno J, Song X, Shi K, Hu W, Mao W, et al (2008) Brassinosteroids Alleviate Heat-Induced Inhibition of Photosynthesis by Increasing Carboxylation Efficiency and Enhancing Antioxidant Systems in *Lycopersicon esculentum*. J. Plant Growth Regul. 27: 49–57.

[16] Hozayn M, Abd El-Monem AA (2010) Alleviation of the potential impact of climate change on wheat productivity using arginine under irrigated Egyptian agriculture. Opions Méditerranéennes. 95: 95–100.

[17] Ko CB, Woo YM, Lee DJ, Lee MC, Kim CS (2007) Enhanced tolerance to heat stress in transgenic plants expressing the GASA4 gene. Plant Physiol Biochem 45: 722–728. 17761429 10.1016/j.plaphy.2007.07.010

[18] Shi WM, Muramoto Y, Ueda A, Takabe T (2001) Cloning of peroxisomal ascorbate peroxidase gene from barley and enhanced thermotolerance by overexpressing in Arabidopsis thaliana. Gene 273: 23–27. 11483357 10.1016/s0378-1119(01)00566-2

[19] Tang L, Kwon SY, Kim SH, Kim JS, Choi JS, et al (2006) Enhanced tolerance of transgenic potato plants expressing both superoxide dismutase and ascorbate peroxidase in chloroplasts against oxidative stress and high temperature. Plant Cell Rep 25: 1380–1386. 16841217 10.1007/s00299-006-0199-1

[20] Alia, Hayashi H, Sakamoto A, Murata N (1998) Enhancement of the tolerance of *Arabidopsis* to high temperatures by genetic engineering of the synthesis of glycinebetaine. Plant J 16: 155–161. 9839462 10.1046/j.1365-313x.1998.00284.x

[21] Yang X, Liang Z, Lu C (2005) Genetic engineering of the biosynthesis of glycinebetaine enhances photosynthesis against high temperature stress in transgenic tobacco plants. Plant Physiol 138: 2299–2309. 16024688 10.1104/pp.105.063164 PMC1183416

[22] Murakami Y, Tsuyama M, Kobayashi Y, Kodama H, Iba K (2000) Trienoic fatty acids and plant tolerance of high temperature. Science 287: 476–479. 10642547 10.1126/science.287.5452.476

[23] Zhang M, Barg R, Yin M, Gueta-Dahan Y, Leikin-Frenkel A, et al (2005) Modulated fatty acid desaturation via overexpression of two distinct omega-3 desaturases differentially alters tolerance to various abiotic stresses in transgenic tobacco cells and plants. Plant J 44: 361–3. 16236147 10.1111/j.1365-313X.2005.02536.x

[24] Qin D, Wu H, Peng H, Yao Y, Ni Z, et al (2008) Heat stress-responsive transcriptome analysis in heat susceptible and tolerant wheat (Triticum aestivum L.) by using Wheat Genome Array. BMC Genomics 9: 432. 18808683 10.1186/1471-2164-9-432 PMC2614437

[25] Smolen GA, Pawlowski L, Wilensky SE, Bender J (2002) Dominant alleles of the basic helix-loop-helix transcription factor ATR2 activate stress-responsive genes in Arabidopsis. Genetics 161: 1235–1246. 12136026 10.1093/genetics/161.3.1235 PMC1462177

[26] Jin H, Martin C (1999) Multifunctionality and diversity within the plant MYB-gene family. Plant Mol Biol 41: 577–585. 10645718 10.1023/a:1006319732410

[27] Stracke R, Werber M, Weisshaar B (2001) The R2R3-MYB gene family in Arabidopsis thaliana. Curr Opin Plant Biol 4: 447–456. 11597504 10.1016/s1369-5266(00)00199-0

[28] Du H, Zhang L, Liu L, Tang XF, Yang WJ, et al (2009) Biochemical and molecular characterization of plant MYB transcription factor family. Biochem (Mosc) 74: 1–11. 10.1134/s0006297909010015 19232042

[29] Mellway RD, Tran LT, Prouse MB, Campbell MM, Constabel CP (2009) The wound-, pathogen-, and ultraviolet B-responsive MYB134 gene encodes an R2R3 MYB transcription factor that regulates proanthocyanidin synthesis in poplar. Plant Physiol 150: 924–941. 19395405 10.1104/pp.109.139071 PMC2689947

[30] Lin-Wang K, Bolitho K, Grafton K, Kortstee A, Karunairetnam S, et al (2010) An R2R3 MYB transcription factor associated with regulation of the anthocyanin biosynthetic pathway in Rosaceae. BMC Plant Biol 10: 50. 20302676 10.1186/1471-2229-10-50 PMC2923524

[31] Mu RL, Cao YR, Liu YF, Lei G, Zou HF, et al (2009) An R2R3-type transcription factor gene AtMYB59 regulates root growth and cell cycle progression in Arabidopsis. Cell Res 19: 1291–1304. 19581938 10.1038/cr.2009.83

[32] Yang Y, Klessig DF (1996) Isolation and characterization of a tobacco mosaic virus-inducible myb oncogene homolog from tobacco. Proc Natl Acad Sci U S A 93: 14972–14977. 8962166 10.1073/pnas.93.25.14972 PMC26247

[33] Sugimoto K, Takeda S, Hirochika H (2000) MYB-related transcription factor NtMYB2 induced by wounding and elicitors is a regulator of the tobacco retrotransposon Tto1 and defense-related genes. Plant Cell 12: 2511–2528. 11148294 10.1105/tpc.12.12.2511 PMC102234

[34] Lee MW, Qi M, Yang Y (2001) A novel jasmonic acid-inducible rice myb gene associates with fungal infection and host cell death. Mol Plant Microbe Interact 14: 527–535. 11310740 10.1094/MPMI.2001.14.4.527

[35] Raffaele S, Vailleau F, Leger A, Joubes J, Miersch O, et al (2008) A MYB transcription factor regulates very-long-chain fatty acid biosynthesis for activation of the hypersensitive cell death response in Arabidopsis. Plant Cell 20: 752–767. 18326828 10.1105/tpc.107.054858 PMC2329921

[36] Feng C, Andreasson E, Maslak A, Mock HP, Mattsson O, et al (2004) Arabidopsis MYB68 in development and responses to environmental cues. Plant Sci 167: 1099–1107.

[37] Negrotto D, Jolley M, Beer S, Wenck A, Hansen G (2000) The use of phosphomannose-isomerase as a selectable marker to recover transgenic maize plants (Zea mays L.) via Agrobacterium transformation. Plant Cell Rep 19: 798–803. 10.1007/s002999900187 30754872

[38] Rogers SO, Bendich AJ (1985) Extraction of DNA from milligram amounts of fresh, herbarium and mummified plant tissues. Plant Mol Biol 5: 69–76. 24306565 10.1007/BF00020088

[39] Miki D, Itoh R, Shimamoto K (2005) RNA silencing of single and multiple members in agene family of rice. Plant Physiol 138(4): 1903–1913. 16172097 10.1104/pp.105.063933 PMC1183382

[40] Livak KJ, Schmittgen TD (2001) Analysis of relative gene expression data using real-time quantitative PCR and the 2(-Delta Delta C(T)) Method. Methods 25: 402–408. 11846609 10.1006/meth.2001.1262

[41] Rosen H (1957) A modified ninhydrin colorimetric analysis for amino acids. Arch. Biochem Biophys 67: 10–15. 10.1016/0003-9861(57)90241-2 13412116

[42] Abrahám E, Hourton-Cabassa C, Erdei L, Szabados L (2010) Methods for determination of proline in plants. Methods Mol Biol 639: 317–31. 20387056 10.1007/978-1-60761-702-0_20

[43] Guijin Z, Bown AW (1997) The rapid determination of g-aminobutyric acid. Phytochem 44: 1007–1009.

[44] Holden HM, Thoden JB, Raushel FM (1998) Carbamoyl phosphate synthetase: a tunnel runs through it. Curr Opin Struc Biol 8: 679–685. 10.1016/s0959-440x(98)80086-9 9914247

[45] Hiroshi U (2000) Enzymatic and structural aspects on glutamate decarboxylase. Journal of Molecular Catalysis B: Enzymatic 10: 67–79.

[46] Du Z, Zhou X, Ling Y, Zhang ZH, Su Z (2010) agriGO: a GO analysis toolkit for the agricultural community. Nucleic Acids Res 38: W64–W70. 20435677 10.1093/nar/gkq310 PMC2896167

[47] Kinnersley AM, Turano FJ (2000) Gamma Aminobutyric Acid (GABA) and Plant Responses to Stress. Crit Rev Plant Sci 19: 479–509.

[48] Alcazar R, Marco F, Cuevas J, Patron M, Ferrando A, et al (2006) Involvement of polyamines in plant response to abiotic stress. Biotechnol Lett 28: 1867–1876. 17028780 10.1007/s10529-006-9179-3

[49] Cheng L, Zou Y, Ding S, Zhang J, Yu X, et al (2009) Polyamine Accumulation in Transgenic Tomato Enhances the Tolerance to High Temperature Stress. J Integrative Plant Biol 51: 489–499. 10.1111/j.1744-7909.2009.00816.x 19508360

[50] Wahid A, Gelani S, Ashraf M, Foolad MR (2007) Heat tolerance in plants: An overview. Environmental and Experimental Botany 61: 199–223.

[51] Rivero RM, Ruiz JM, Romero LM (2004) Importance of N source on heat stress tolerance due to the accumulation of proline and quaternary ammonium compounds in tomato plants. Plant Biol (Stuttg) 6: 702–707. 15570475 10.1055/s-2004-821293

[52] Szabados L, Savouré A (2010) Proline: a multifunctional amino acid. Trends in Plant Science 15: 89–97. 20036181 10.1016/j.tplants.2009.11.009

[53] Morris SM (2007) Arginine Metabolism: Boundaries of Our Knowledge. J Nutr 137: 1602S–1609S. 17513435 10.1093/jn/137.6.1602S

[54] Wen XP, Pang XM, Matsuda N, Kita M, Inoue H, et al (2008) Over-expression of the apple spermidine synthase gene in pear confers multiple abiotic stress tolerance by altering polyamine titers. Transgenic Res. 17: 251–263. 10.1007/s11248-007-9098-7 17549601

[55] Becker T, Fock H (1986) The activity of nitrate reductase and the pool sizes of some amino acids and some sugars in water-stressed maize leaves. Photosynth Res 8: 267–274. 24443264 10.1007/BF00037134

