# Supplementary material for: The Rice R2R3-MYB Transcription Factor OsMYB55 Is Involved in the Tolerance to High Temperature and Modulates Amino Acid Metabolism
Source: PLoS One. 2012 Dec 14;7(12):e52030. doi: 10.1371/journal.pone.0052030 (PMC3522645; doi:10.1371/journal.pone.0052030)
Supplement: Figure S1 — Rice panicles as affected by high temperature. High temperature inhibits panicle length (A). High temperature caused panicle and floret deformation at flowering stage when plants were grown under long day (C) or neutral day (D) compared to the panicle of rice plants grown at moderate temperature (B). Failure of grain filing at harvest stage in the plants grown at 35°C under long day (F) or neutral day (G) compared to the plants grown at moderate temperature (E). (PDF) [file pone.0052030.s001.pdf]

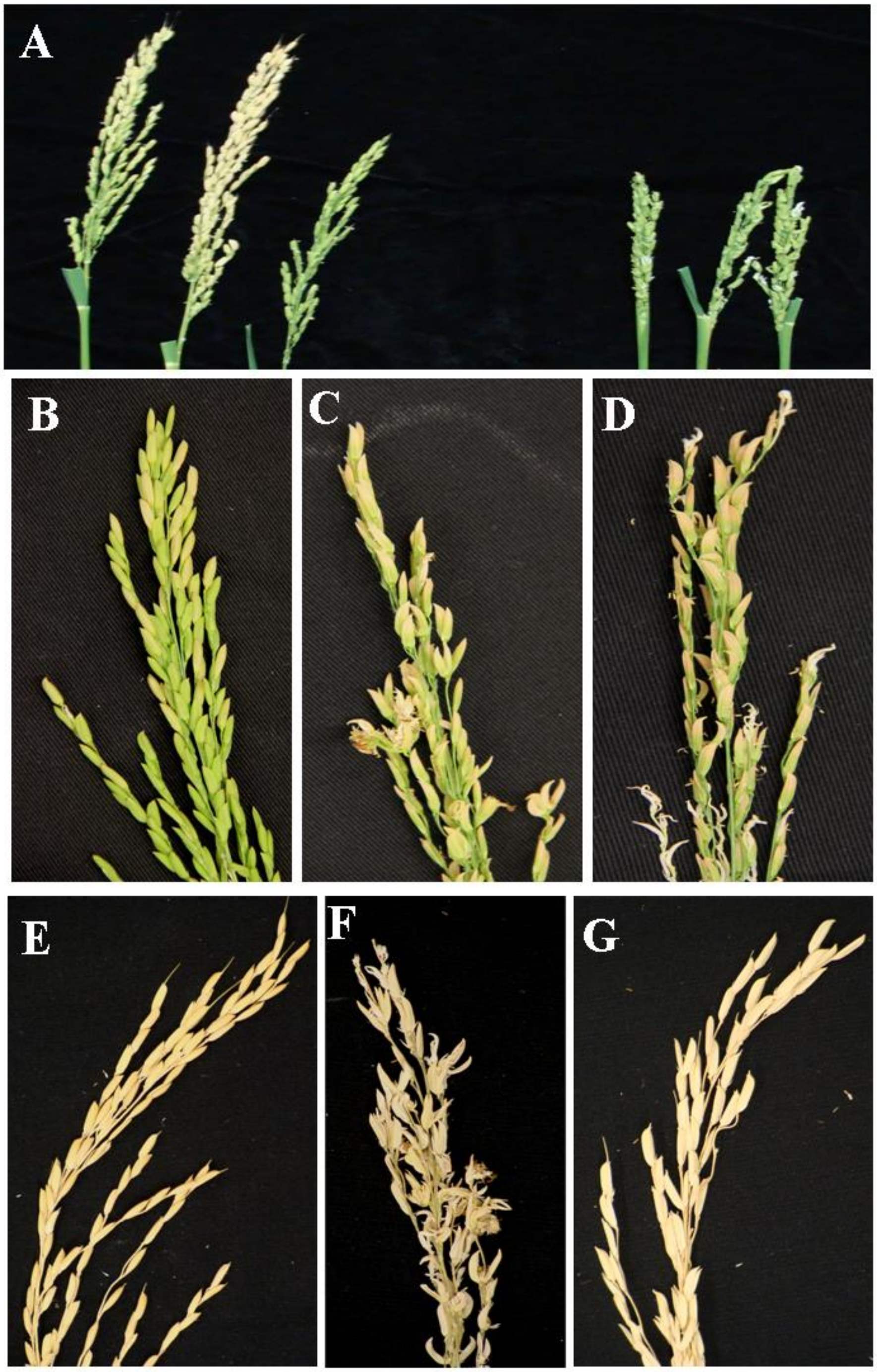

Figure S1. Rice panicles as affected by high temperature. High temperature inhibits panicles length (**A**). High temperature caused panicle and floret deformation at flowering stage when plants were grown under long day (**C**) or neutral day (**D**) compared to the panicle of rice plants grown at normal temperature (**B**). Failure of grain filing at harvest stage in the plants grown at 35°C under long day (**F**) or neutral day (**G**) compared to the plants grown at normal temperature (**E**).
