# Supplementary material for: The Rice R2R3-MYB Transcription Factor OsMYB55 Is Involved in the Tolerance to High Temperature and Modulates Amino Acid Metabolism
Source: PLoS One. 2012 Dec 14;7(12):e52030. doi: 10.1371/journal.pone.0052030 (PMC3522645; doi:10.1371/journal.pone.0052030)
Supplement: Figure S2 — Transcriptome changes between the wild type and transgenic plants upon the exposure to high temperature. The Venn diagrams represent the number of genes that were found to be significantly differentially expressed between OsMYB55 overexpression plants and wild-type (WT) in response to high temperature treatment. Four weeks old rice plants were exposed to high temperature (45°C) for one hour. The upper Venn diagram (A) represents the number of genes that were found to be up-regulated in the transgenic and wild plants in response to high temperature, and the lower Venn diagram (B) shows the number of genes that were found to be down-regulated. Genes in the medium are regulated in both transgenic and wild type plants. (PDF) [file pone.0052030.s002.pdf]

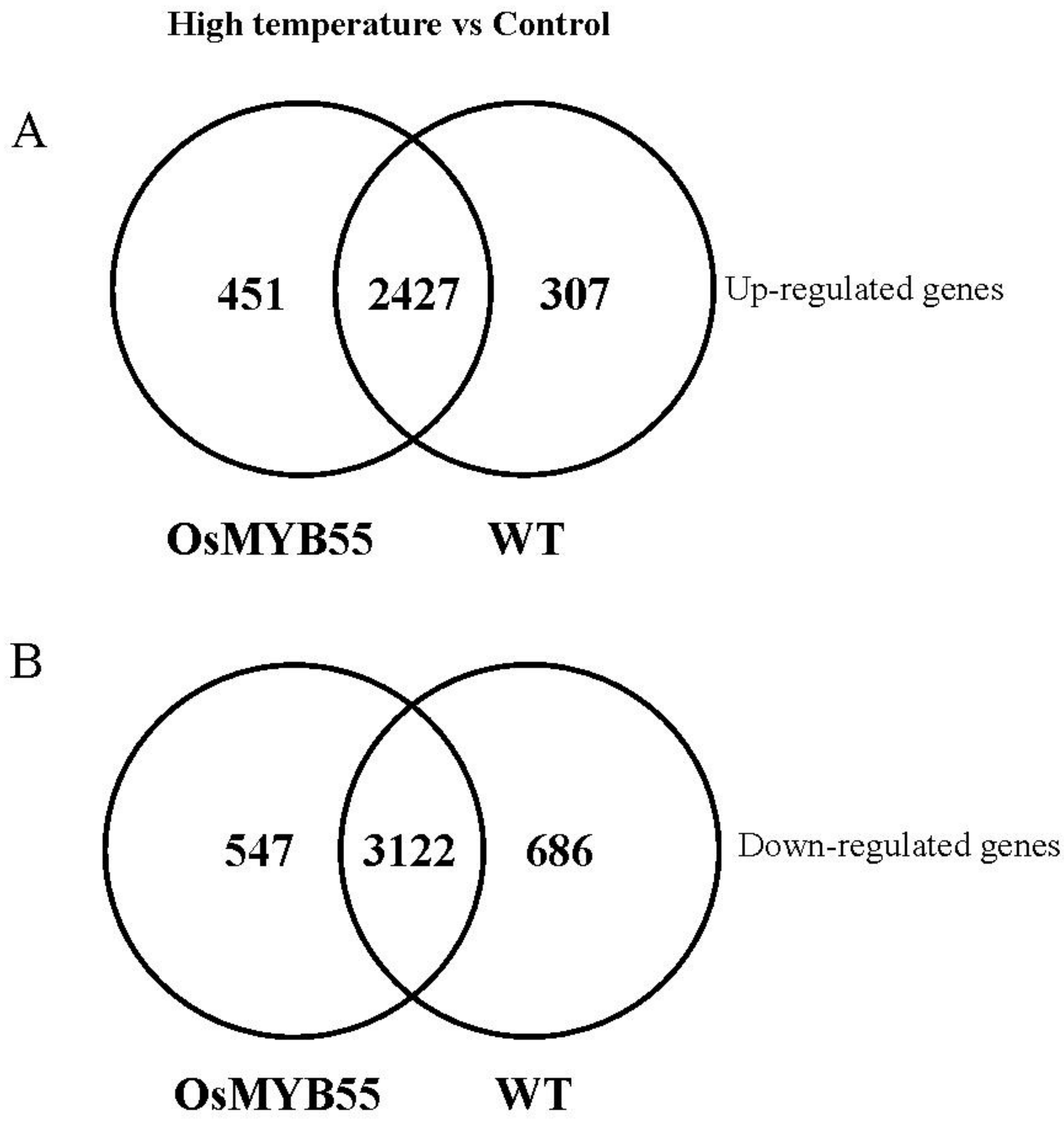

Figure S2. Transcriptome changes between the wild type and transgenic plants upon the exposure to high temperature. The Venn diagrams represent the number of genes that were found to be significantly differentially expressed between OsMYB55 overexpression plants and wild-type (WT) in response to high temperature treatment. Four weeks old rice plants were exposed to high temperature (45 °C) for one hour. The upper Venn diagram (**A**) represents the number of genes that were found to be up-regulated in the transgenic and wild plants in response to high temperature, and the lower Venn diagram (**B**) shows the number of genes that were found to be down-regulated. Genes in the medium are regulated in both transgenic and wild type plants.
