# Supplementary material for: The Rice R2R3-MYB Transcription Factor OsMYB55 Is Involved in the Tolerance to High Temperature and Modulates Amino Acid Metabolism
Source: PLoS One. 2012 Dec 14;7(12):e52030. doi: 10.1371/journal.pone.0052030 (PMC3522645; doi:10.1371/journal.pone.0052030)
Supplement: Table S1 — Significantly enriched upregulated biological processes in the wild type and Myb transgenic plants during heat treatment (PDF) [file pone.0052030.s003.pdf]

Table S1: Significantly enriched upregulated biological processes in the wild type and Myb transgenic plants

GO Term	Onto	Description	wt	Myb
GO:0006457	P	protein folding		
GO:0050896	P	response to stimulus		
GO:0006950	P	response to stress		
GO:0009266	P	response to temperature stimulus		
GO:0010035	P	response to inorganic substance		
GO:0009644	P	response to high light intensity		
GO:0009642	P	response to light intensity		
GO:0034641	P	cellular nitrogen compound metabolic process		
GO:0009408	P	response to heat		
GO:0008380	P	RNA splicing		
GO:0009628	P	response to abiotic stimulus		
GO:0042542	P	response to hydrogen peroxide		
GO:0000041	P	transition metal ion transport		
GO:0033036	P	macromolecule localization		
GO:0045184	P	establishment of protein localization		
GO:0015031	P	protein transport		
GO:0008104	P	protein localization		
GO:0065009	P	regulation of molecular function		

during heat treatment

wt		Myb	
FDR	Num	FDR	Num
1.20E-15	42	8.90E-15	42
1.10E-06	136	3.00E-06	140
7.10E-06	90	2.20E-05	92
1.30E-05	22	2.90E-05	22
5.90E-05	15	2.30E-05	16
0.00012	8	0.00019	8
0.0011	9	0.0016	9
0.0013	49	0.00069	52
0.002	10	0.0033	10
0.0068	11	0.0098	11
0.013	40	0.0051	43
0.028	7	0.03	7
0.028	10	---	---
0.037	32	0.0054	36
---	---	0.018	33
---	---	0.018	33
---	---	0.028	33
---	---	0.04	14
